# Zika Virus Infects Intermediate Progenitor Cells and Post-mitotic Committed Neurons in Human Fetal Brain Tissues

**DOI:** 10.1038/s41598-017-13980-2

**Published:** 2017-11-01

**Authors:** Ming-Yi Lin, Yi-Ling Wang, Wan-Lin Wu, Victoria Wolseley, Ming-Ting Tsai, Vladimir Radic, Matthew E. Thornton, Brendan H. Grubbs, Robert H. Chow, I-Chueh Huang

**Affiliations:** 1 Department of Physiology & Biophysics, Keck School of Medicine, University of Southern California, Los Angeles, CA USA; 2 Department of Molecular Microbiology and Immunology, Keck School of Medicine, University of Southern California, Los Angeles, CA USA; 3 Department of Obstetrics and Gynecology, Keck School of Medicine, University of Southern California, Los Angeles, CA USA

## Abstract

Zika virus (ZIKV) infection is associated with microcephaly in fetuses, but the pathogenesis of ZIKV-related microcephaly is not well understood. Here we show that ZIKV infects the subventricular zone in human fetal brain tissues and that the tissue tropism broadens with the progression of gestation. Our research demonstrates also that intermediate progenitor cells (IPCs) are the main target cells for ZIKV. Post-mitotic committed neurons become susceptible to ZIKV infection as well at later stages of gestation. Furthermore, activation of microglial cells, DNA fragmentation, and apoptosis of infected or uninfected cells could be found in ZIKV-infected brain tissues. Our studies identify IPCs as the main target cells for ZIKV. They also suggest that immune activation after ZIKV infection may play an important role in the pathogenesis of ZIKV-related microcephaly.

## Introduction

The association between ZIKV and microcephaly in fetuses has triggered a global public health emergency^1^. Since the initial isolation of ZIKV in 1947, ZIKV has spread from Africa to Asia, Oceania, and Latin America^2–5^. ZIKV, a member of flaviviruses, is closely related to dengue virus (DENV) and yellow fever virus^4,6^. Flavivirus envelope (E) proteins are responsible for receptor association and viral entry^7^. Several viral nonstructural (NS) proteins are essential for viral replication and associated with pathogenesis^8^. The structure of ZIKV E protein has been reported and several neutralizing antibodies targeting ZIKV E protein that potentially have therapeutic applications, have also been isolated^7,9,10^. Clinical presentations of ZIKV fever are usually mild including fever, headaches, maculopapular rash, malaise, conjunctivitis, myalgia and arthralgia^4^. Neurological manifestations, including Guillain–Barré syndrome, were initially reported during the ZIKV outbreak in French Polynesia. Not until the 2015–2016 epidemic of ZIKV in Brazil was the association between ZIKV and microcephaly in human fetuses established^1,11–13^.

Brain cortical development starts at gestational week (GW) 5 in humans^14,15^. Several neural progenitor cells can be found during the processes of neurogenesis. Neuroepithelial cells (NECs) and radial glial cells (RGCs) localized to the ventricular zone exhibit stem cell properties and express stem cell markers, including sex determining region Y-box 2 (SOX2) and nestin^14,16,17^. In contrast, intermediate progenitor cells (IPCs) are mainly distributed in the subventricular zone and undergo limited mitotic division^18–21^. Although both RGCs and IPCs differentiate into neurons, IPCs generate most of the excitatory neurons in the cortical plate (80% of excitatory pyramidal neurons)^22,23^. T-box brain protein 2 (Tbr2), which is expressed uniquely in IPCs, is commonly used to label IPCs, whereas T-box brain protein 1(TBR1), special AT-rich sequence-binding protein 2 (SATB2), and microtubule-associated protein 2 (MAP2) are specific markers for post-mitotic differentiated neurons^19,24–26^. In addition to cells of the neuronal lineage, astroglial and oligodendroglial cells are important cellular components in developed brains. Recent studies have shown that glial cells are detected in the early stages of brain development^27–30^. Nevertheless, gliogenesis is more active in the third trimester of gestation^31^.

ZIKV has been isolated from brain tissues of newborns with microcephaly^1^. A number of groups have addressed the question of which cell type might be targeted by using induced pluripotent stem cells (iPSCs) or embryonic stem cells (ESCs) as model systems. Their studies have concluded that ZIKV infects neural progenitor cells derived from induced pluripotent stem cells (iPSCs) or embryonic stem cells (ESCs)^32–35^. It impairs neurosphere survival and growth of iPSC/ESC-derived brain organoids^32,36^. However, the heterogeneous gene expression profiles of iPSCs and ESCs and the lack of normal brain architecture in iPSC and ESC model systems raise questions about their validity for modeling brain infection^37^. Recently, infection of ZIKV in RGCs in human fetal brain tissues has been reported^38^. A limited number of brain slices were analyzed and the percentage of ZIKV-infected cells expressing the RCG marker was extremely low suggesting that principal target cells for ZIKV infection were yet to be identified. To better understand the pathogenesis of ZIKV-related microcephaly, we investigated the tissue and cellular tropism of ZIKV in human fetal brain tissues.

## Results

### ZIKV infects IPCs and post-mitotic committed neurons

**Figure 1:**
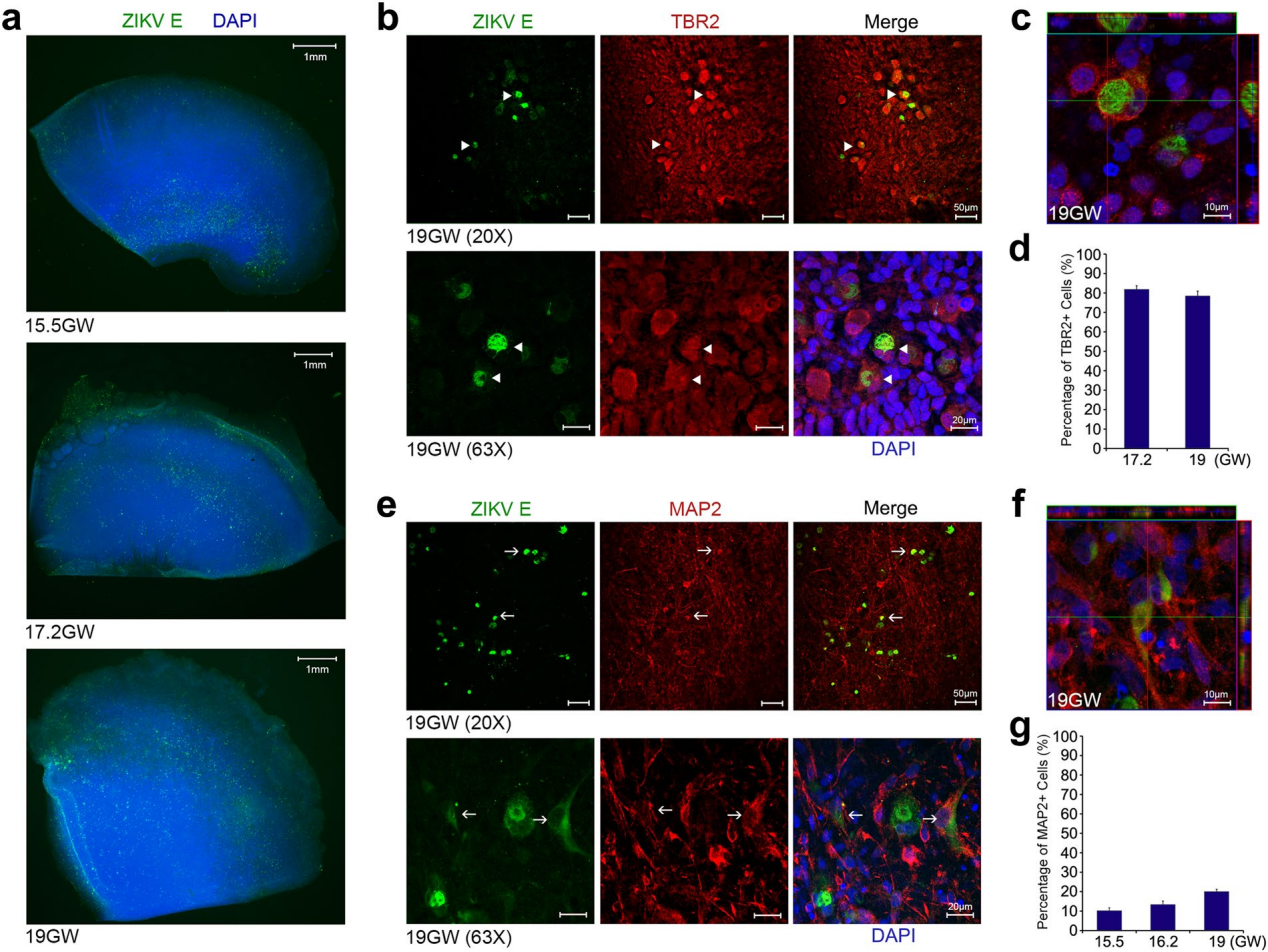
ZIKV infects IPCs and post-mitotic committed neurons. (**a**) Human fetal brain tissues from donors at the indicated gestation ages were infected with 1 × 10^6^ plaque-forming unit (PFU) ZIKV strain MR766. Two days later, tissues were fixed, permeabilized, labeled with an anti-flavivirus E antibody and 4′,6-diamidino-2-phenylindole (DAPI). Labeled cells were imaged by fluorescent microscopy. (**b**) Experiments were similar to those in (**a**) except that infected tissues were labeled with the indicated antibodies and imaged by confocal microcopy. (**c**) Experiments were similar to those in (**b**) except that x-y, y-z, and x-z axis images are show. Arrowheads indicate ZIKV-infected Tbr2-positive cells. (**d**) Quantitative analysis of percentages of Tbr2-positive cells among ZIKV-infected cells in fetal brain tissues at the indicated ages. (**e**) Experiments were similar to those in (**a**,**b**) except that the indicated antibodies were used. (**f**) Experiments were similar to those in (**e**) except that x-y, y-z, and x-z axis images are show. Arrows indicate ZIKV-infected MAP2-positive cells. (**g**) Quantitative analysis of the percentage of MAP2-positive cells among ZIKV-infected cells in fetal brain tissues at the indicated ages. (**d**,**g**) More than 100 infected cells were examined for each counting. The procedures were repeated for 3 times for each sample at the indicated ages. 10–20 fields for each brain slice have been inspected to draw our conclusions. Error bars denote one s.d. (*n* = 3).

Human fetal brain tissues from 14–21 GW donors were thinly sliced and infected with ZIKV strain MR766 (Extended Data Table 1). Our studies were confined to fetal brain tissue in the second trimester due to the lack of accessibility to brains tissues from donors in the first and third trimesters of gestation. Infected tissues were labeled with an antibody targeting ZIKV E protein. In fetal brain tissues from the 15.5 GW donor, we observed that the subventricular zone was highly susceptible, whereas the intermediate zone and the cortical plate were less permissive to ZIKV infection. ZIKV tissue tropism broadens at later stages of gestation (Fig. 1a and Extended Data Figs 1 and 2a,b). From mid-second trimester, ZIKV E protein could also be detected in the intermediate zone and the cortical plate. Infection of ZIKV in the ventricular zone, where neural stem cells are clustered, was not substantial across the ages we examined.

Radial migration of neuronal cells from the ventricle to the dorsal side of fetal brains is the landmark feature of mammalian neurogenesis^39,40^. The similar migration pattern of ZIKV tissue tropism raises the possibility that ZIKV may infect cells of the neuronal lineage. To evaluate this possibility, ZIKV MR766-infected fetal brain tissues were labeled with different markers specific to neuronal lineage cells. We observed that Tbr2-expressing cells were high permissive to ZIKV infection indicating that IPCs are the main target cells for ZIKV (Fig. 1b–d and Extended Data Fig. 3a–c). The atypical subcellular distribution of Tbr2 in IPCs might be caused by cytopathic changes due to ZIKV infection. Although colocalization of ZIKV E protein and Ki-67 could be found, this association was not exclusive suggesting that being in a proliferative stage does not determine the susceptibility of cells to ZIKV infection (Extended Data Fig. 3d). In addition to being found in IPCs, ZIKV E protein signal could be detected in MAP2-expressing cells in brain tissues (Fig. 1e,f and Extended Data Fig. 4a–c). The percentage of MAP2-postitive cells in ZIKV-infected cells increased at later stages of gestation (Fig. 1g). In summary, our studies demonstrate that IPCs are main target cells of ZIKV in human fetal brain tissues. Post-mitotic committed neurons increase in their susceptibility to ZIKV infection during the second trimester.

### Neural stem cells in human fetal brain tissues are not main target cells of ZIKV

**Figure 2:**
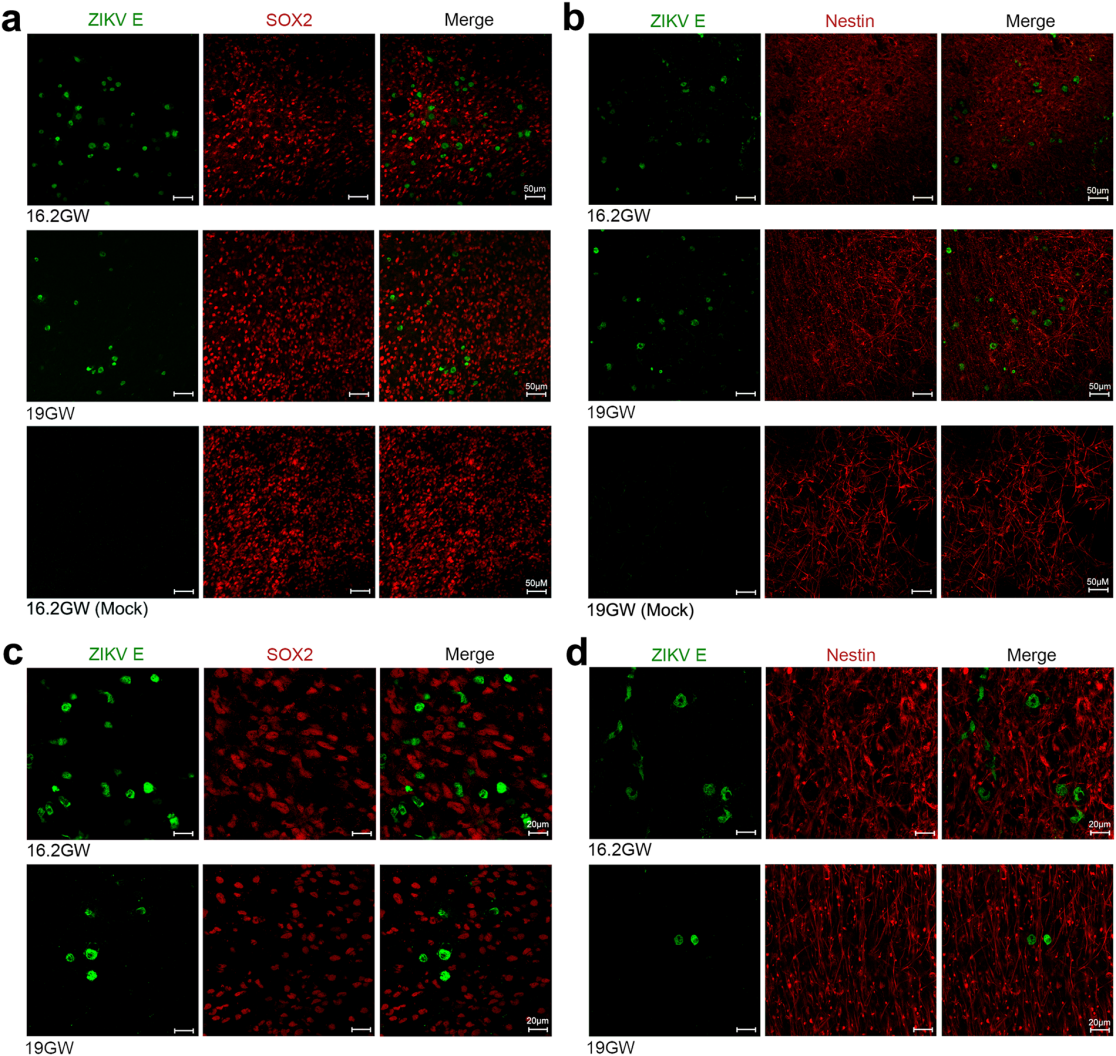
ZIKV does not infect neural stem/progenitor cells in human fetal brain tissues. (**a**,**b**) Human fetal brain tissues from donors at the indicated gestation ages were mock infected or infected with 1 × 10^6^ PFU of ZIKV strain MR766. Two days later, tissues were labeled with the indicated antibodies. Labeled cells were imaged by confocal microscopy. (**c**,**d**) Experiments were similar to those in (**a**,**b**) except that high magnification images are shown. 10–20 fields and more than 300 ZIKV-infected cells for each brain slice were inspected to draw our conclusions. Arrows indicate ZIKV-infected cells. Markers used included SOX2 and nestin for neural stem cells.

Recent studies using iPSC and ESC systems have demonstrated that ZIKV infects SOX2- and nestin-expressing cells suggesting that neural stem cells, such as NECs and RGCs, are target cells of ZIKV^32,36^. Colocalization of ZIKV NS1 and vimentin (VIM), a RGC marker, in brain slices from a healthy fetus was also reported^38^. To examine whether neural progenitor cells with stem cell properties in human fetal brain tissues are similarly susceptible to ZIKV infection, brain slices were infected with ZIKV and labeled for different stem cell markers. We inspected multiple samples from donors at different gestational ages but could not find substantial colocalization of ZIKV E and SOX2 or nestin signals (Fig. 2a–d). These findings suggested that neural stem cells are not major target cells in fetal brain tissues.

Unlike the case in rodents, gliogenesis in humans starts in the early stages of gestation^27,31^. We further included anti-neural/glial antigen 2 (NG2), a oligodendroglial marker^30^, and anti-glial fibrillary acidic protein (GFAP), which is expressed in RGCs and astroglial cells in humans^41,42^, antibodies in our studies. Although ZIKV E signal could be detected in only few GFAP- or NG2-expressing cells among the sample examined (Extended Data Fig. 5a–d), the significance of these observations is unclear. The populations of astroglial and oligodendroglial cells are small in the early stages of gliogenesis^31^. We could not exclude the possibility that these cells may play a more important role in the pathogenesis of ZIKV-related microcephaly in the third trimester of gestation.

The discrepant observations between previous publications and our work raised the concern that different model systems may alter the apparent ZIKV tropism. To examine this possibility, similar infection assays were performed in neural monolayers derived from brain tissues. Compatible with previous reports, we found that ZIKV signal could be detected in SOX2-, GFAP-, and nestin-expressing cells (Extended Data Fig. 6a,b)^32,35,36^. Collectively, our data indicate that the multicellular architecture and the complex microenvironment of the brain are important determinants contributing to the tropism of ZIKV. They also suggest that a more physiologically relevant system is needed for *in vitro* ZIKV research.

### ZIKV infection activates innate immune responses in human fetal brain tissues

**Figure 3:**
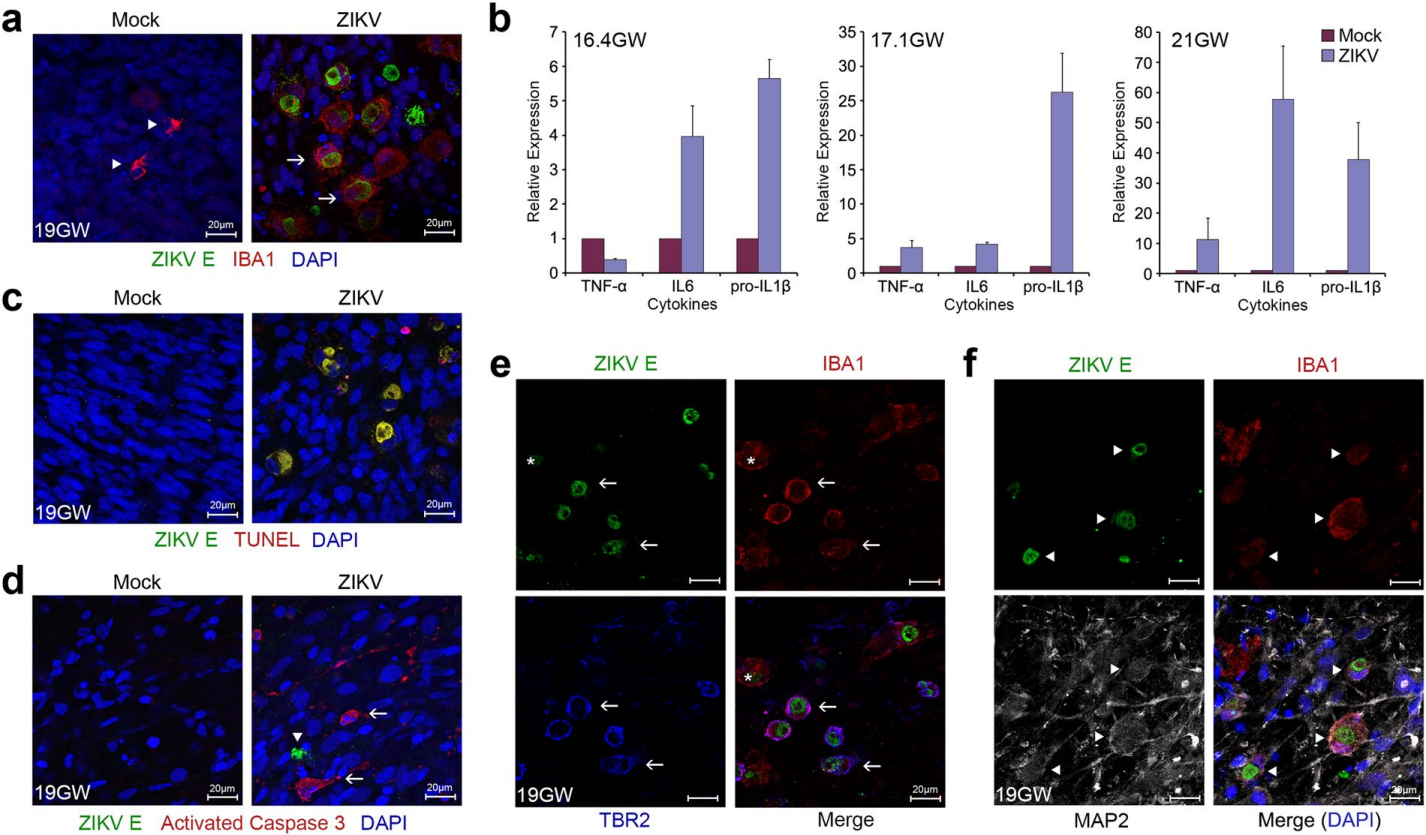
ZIKV infection activates innate immune responses in human fetal brain tissues. (**a**) Human fetal brain tissues from donors at the indicated gestation ages were mock infected or incubated with 1 × 10^6^ PFU ZIKV strain MR766. Two days later, tissues were fixed, permeabilized, and labeled with the indicated antibodies. Labeled cells were imaged by confocal microcopy. Arrowheads indicate resting and arrows indicate activated microglial cells. (**b**) Experiments were similar to those in (**a**) except that expression of the indicated cytokines was assayed by qRT-PCR. Cytokine expression of infected tissues relative to that of uninfected controls is shown. Error bars denote one s.d. (*n = 3*). *P < 0.05 compared with controls. (**c**) Experiments were similar to those in (**a**) except that terminal deoxynucleotidyl transferase dUTP nick end labeling (TUNEL), instead of Iba-1 labeling, was used. (**d**) Experiments were similar to those in (**a**) except that an anti-activated caspase 3, instead of anti-Iba-1, antibody was used to label tissues. Arrowheads indicate ZIKV-infected cells and arrows indicate uninfected cells. (**e**,**f**) Experiments were similar to those in (**a**) except that the indicated antibodies were used to label ZIKV-infected tissues. Arrows indicate colocalization of Tbr2 and Iba1 in ZIKV-infected cells. Arrowheads indicate colocalization of MAP2 and Iba1 in ZIKV-infected cells. *Indicates the Iba1-positive but Tbr2-negative cell.

Activation of the caspase pathway in iPSC-derived neural progenitor cells after ZIKV infection has recently been reported^32^. We subsequently examined innate immune profiles of ZIKV-infected human fetal brain tissues. We observed that ZIKV infection triggered elevated expression of proinflammatory cytokines and activation of microglial cells (Fig. 3a,b and Extended Data Fig. 7a–c). In addition, DNA fragmentation could be found in almost every single infected cell and activated caspase 3, a marker for activation of apoptosis, could be detected in many uninfected cells (Fig. 3c,d and Extended Data Fig. 8a,b). These findings suggest that expression of inflammatory cytokines triggered by ZIKV infection may have direct or indirect cytopathic effects on uninfected cells. In our experiments, we also noticed that ionized calcium-binding adapter molecule 1 (Iba1), a marker for microglial cells, partially colocalized with Tbr2 or MAP2 in ZIKV-infected cells (Fig. 3e,f and Extended Data Fig. 9a–c). Microglia have been shown to regulate neurogenesis late in brain development in primates^43^. Proinflammatory cytokines secreted by activated microglial cells have also been reported to suppress neurogenesis^44^. Our data indicate that immune reactions may also play an important role in the pathogenesis of ZIKV-related microcephaly.

## Discussion

IPCs contribute to neurogenesis of fetal brains^18,45^. Depletion of IPCs by mutating genes essential for IPC differentiation and proliferation is associated with microcephaly in animals^18^. Silencing expression of Tbr2, which regulates gene expression in IPCs, similarly leads to microcephaly in humans^46^. Susceptibility of IPCs and post-mitotic committed neurons to ZIKV infection and activation of microglial cells observed in our research indicate that infection of these cells may play a pivotal role in the pathogenesis of ZIKV-related microcephaly. One possible mechanism for ZIKV-related microcephaly is that ZIKV infection results in depletion of IPCs and differentiating neurons. Inflammatory reactions triggered by ZIKV infection may induce apoptosis of uninfected cells and worsen cell loss in developing brains.

Onorati *et al*. reported that ZIKV infects RGCs in fetal brain tissues from a healthy fetus^38^. Although colocalization between ZIKV NS1 and VIM could be found, the majority of ZIKV-infected cells, which did not colocalize with VIM, has not been characterized. In addition, similar phenotypes could not be observed in brain tissues from a ZIKV-infected fetus. ZIKV NS1, used in their experiments, is a secreted viral protein. Previous studies have shown that DENV NS1 interacts with several cellular glycoproteins at the plasma membrane^47^. It is not clear whether the ZIKV NS1 signal detected was from infected cells or from the interaction between NS1 and surface molecules of uninfected cells. In our studies, we comprehensively characterized the tropism of ZIKV in human fetal brain tissues and observed that IPCs are the principal target cells for ZIKV, whereas neural progenitor cells with stem cells properties are not susceptible to ZIKV infection.

The iPSC/ESC system and their derived brain organoids have been widely used for research of ZIKV-associated microcephaly^32–35^. The susceptibility of neural stem cells derived from iPSCs/ESCs, but not those in human fatal brain slices, to ZIKV infection indicates that properties of neural system cells in these two systems may have different properties. It also suggests that human fetal brain tissues are a more physiologically relevant *ex vivo* model for ZIKV-related studies. Although fetal brain slices preserve most of the cell population and the architecture of developing brains, the lack of adaptive immune cells and an intact blood brain barrier limits its research applications. Further *in vivo* investigations into central nervous system transmission and immune responses after ZIKV infection are warranted.

## Methods

### Human Subjects

All methods for human subject-related studies in this work were carried out in accordance with Federal Policy for the Protection of Human Subjects and University of Southern California Human Subjects Protection Program (HSPP) Policies and Procedures. All experimental protocols were approved by the institutional review board of University of Southern California. Informed consent was obtained from all subjects.

### Brain slice preparation and organotypical culture

Postmortem fetal brain tissues were donated by18 donors (Extended Data Table 1). Specimens were transported in Hypothermosol solution (Sigma, USA; Biolife Solutions, USA) on ice and the transport time was limited to under two hours. Brain tissues were visually examined for structural integrity. The anterior or posterior face of the tissue was glued onto specimen plate and sliced into 400 μm slices in cold (4 °C), oxygenated (95% O_2_, 5% CO_2_) N-methyl-D-glucamine-artificial cerebral spinal fluid (NMDG-ACSF: NMDG 93 mM; KCl 2.5 mM; NaH_2_PO_4_ 1.2 mM; NaHCO_3_ 30 mM; HEPES 20 mM; Glucose 25 mM; Sodium ascorbate 5 mM; Thiourea 2 mM; Sodium pyruvate 3 mM; MgSO_4_ 10 mM; CaCl_2_ 0.5 mM) using a vibratome (Leica VT1200S, Germany). Vertical deflection of the blade was minimized with Vibrocheck technology (The slicing speed was 0.1–0.15 mm/s and the vibration amplitude was1.5 mm). Slices were transferred to a recovery chamber (32 °C, 95% O_2_, 5% CO_2_) and allowed to recover in ACSF (NaCl 124 mM; KCl 4 mM; NaHCO_3_ 26 mM; Glucose 10 mM; CaCl_2_ 2 mM; MgCl_2_ 2 mM) for 30–60 minutes before placing into inserts (Millipore Bioscience Research Reagents) for culture. Tissues were maintained in Dulbecco’s Modified Eagle Medium (DMEM glutamax; ThermoFisher Scientific) supplemented with 10% fetal bovine serum (FBS; Corning), 100U/ml penicillin, and 100 μg/ml streptomycin (Corning) with the culture condition of 37 °C and 5% CO_2_. Half of the medium was replaced with fresh medium every other day.

### Human primary brain monolayer culture

Human primary brain monolayers were prepared as described^48^. In brief, visible blood vessels and blood-contaminated areas were removed from 7–15 grams of the brain tissues. Tissues were then chopped into small pieces of approximately 0.5 mm thickness. Tissue masses were incubated with 10 mL of trypsin-EDTA and oscillated at 150 RPM for 10–15 minutes at 37 °C. Tissue was homogenized by trituration with wide-bore glass Pasteur pipettes for 10–12 times and tissue debris was removed with a cell strainer (70 μM, BD Biosciences). Digested cells were washed twice with DMEM and resuspended in defined BrainPhys Neuronal medium (STEMCELL Technologies), supplemented with 1X B27 (STEMCELL Technologies), 1X N2 (STEMCELL Technologies), 50 mM Glutamax (Gibco), 1X pen/strep cocktail (Corning) and recombinant human basic fibroblast growth factor (bFGF) (Invitrogen) at a concentration of 5 ng/mL. Cells were plated in poly-L-lysine-coated plates (200,000 cells/ 24-well). Half of the culture medium was replaced with fresh medium every 4 days.

### ZIKV infection

ZIKV strain MR766 was obtained from ATCC and propagated in Vero cells for 4 days. Viral supernatant was filtered via 0.45 μM syringe filters (VWR). Virus titers were determined by a standard plaque assay as described previously^49,50^. Human fetal brain slice culture was cultured for 4 days before incubating with the indicated PFUs of ZIKV. Two hours later, tissues were washed once with phosphate buffered saline (PBS) and maintained in regular culture medium. Infected tissues were fixed for further image studies 48 hours after infection. For primary human brain monolayer infection, cells were incubated with the indicated PFUs of ZIKV for 2 hours. Cells were then washed with PBS once and maintained in regular medium. Previous studies have demonstrated that high ZIKV titers could be detected in fetal brain tissues, placenta and umbilical cords^11,13^. In these studies, real-time PCR was extensively used to determine viral titers in human maternal and fetal samples. Although the standard plaque assay was used for viral titering, we have standardized ZIKV titers to RNA copy numbers by real-time PCR. The titers use in our studies were comparable to those measured in human fetal brains.

### qRT-PCR

Total RNA from human fetal brain tissues was extracted using an RNeasy Mini Kit (QIAGENE) following the manufacturer’s protocol. qRT-PCR was performed using an iScript One-Step RT-PCR kit and iTaq Universal SYBR Green Supermix (Bio-Rad). The thermal cycling condition included an initial denaturation step at 95 °C for 2 minutes and 40 reaction cycles consisting of a denaturation step at 95 °C for 5 seconds and an annealing/elongation step at 60 °C for 30 seconds. Cytokine expression was normalized to the eukaryotic translation initiation factor 2B (EIF2B2). The relative expression was further calculated using the comparative CT (ΔΔCT) method^51^. The sequences of primers used were as follows:

eIF2B2_QPCR_Forward: 5′ TCCACCCCACTCATCGTCTG3′

eIF2B2_QPCR_Reverse: 5′ TGGCAGGACTTCTTCAGGAGC3′

TNF-α_Forward: 5′AGG CGG TGC TTG TTC CTC A3′

TNF-α_Reverse: 5′GTT CGA GAA GAT GAT CTG ACT GCC3′

IL-6_Forward: 5′CCT TCC AAA GAT GGC TGA AA3′

IL-6_Reverse: 5′ CAG GGG TGG TTA TTG CAT CT3′

Pro-IL1β_Forward: 5′ AGCTACGAATCTCCGACCAC3′

Pro-IL1β_Reverse: 5′CGTTATCCCATGTGTCGAAGAA3′

### Immunofluorescence staining and imaging

Two days after viral inoculation, ZIKV-infected fetal brain slices were washed with PBS three times and fixed with 4% paraformaldehyde (Alfa Aesar) at 4 °C overnight. On the second day, tissues were washed with PBS three times and incubated with staining solution containing 3% BSA (Sigma-Aldrich), 0.2% Triton-X (Sigma-Aldrich) and various primary antibodies. Primary antibodies used included murine anti-pan-flavivirus E protein (Millipore, MAB10216, 1:500), murine anti-ZIKV E protein IgG2b (GeneTex, GTX634155, 1:200), goat anti-sex determining region Y-box 2 (SOX2, Abcam, ab110145, 1:100), rabbit anti-glial fibrillary acidic protein (GFAP, Abcam, ab7260, 1:400), rabbit anti-Ki67 (ThermoFisher, PA5-19462, 1:500), chicken anti-microtubule-associated protein 2 (MAP2, ThermoFisher, PA1-10005, 1:1,000), chicken anti-T-box brain protein 2 (TBR2, Millipore, AB15894, 1:200), rabbit anti-TBR2 (Millipore, AB2283, 1:300), murine anti-TBR2 IgG2b (Millipore, MA5-24291, 1:200), murine anti-SATB2 IgG1 (Santa Cruz Biotechnology, sc-81376, 1:250), rabbit anti-cleaved caspase 3 (Cell Signaling, 9661, 1:100), rabbit anti-Iba1 (WAKO, 019-19741, 1:250), goat anti-Iba1(Abcam, ab5076, 1:250), rat anti-CD11b (Abcam, ab8878, 1:200), rat anti-HLA-DR (Abcam, ab134038, 1:200) and rabbit anti-neural/glial antigen 2 (NG2, Millipore, AB5320, 1:50) antibodies. After primary antibody incubation, slices were washed with PBS three times and secondary antibodies applied for overnight at 4 °C. Secondary antibodies used included Alex 488-conjugated goat anti-mouse IgG1, Alex 647-conjugated goat anti-mouse IgG2b, Alexa 488-conjugated goat anti-mouse IgG IgM, Alexa 488-conjugated donkey anti-mouse IgG, Alexa 488-conjugated goat anti-rat IgG, Alexa 555-conjugated goat anti-mouse IgG, Alexa 555-conjugated goat anti-rabbit IgG, Alexa 555-conjugated donkey anti-rabbit IgG, Alexa 647-conjugated goat anti-chicken IgY, Alexa 633-conjugated donkey anti-chicken IgY and Alexa 647-conjugated donkey anti-goat IgG antibodies (ThermoFisher, 1:1,000). Terminal deoxynucleotidyl transferased UTP Nick End Labeling (TUNEL) assay (Cell Death Detection Kit, Roche) was performed following the manufacturer’s instructions. Antibody-labeled tissues were washed 3 times with PBS, counterstained with 4′,6-diamidino-2-phenylindole (DAPI; ThermoFisher), and mounted with CitiFluor AF1 (Electron Mocroscopy Sciences). Slices were imaged by the BZ-X700 microscope or Leica TCS SP8 or Zeiss confocal microscopes.

### Image and statistical analyses

We analyzed 94 slices from 18 donors in total. For each neural marker, we inspected 10 slices and 10–20 fields for each slice to draw our conclusions. For quantitative analysis, more than 100 ZIKV-infected cells were examined per experiment. The same procedures were repeated at least 3 times. Error bars denote one standard deviation (s.d.) of triplicates. For cytokine studies, we used a paired two-tailed Student’s *t*-test for statistical analyses, and P < 0.05 was considered significant. The data that support the findings of this study are available upon reasonable request.

## Electronic supplementary material

Supplementary Information
