# Supplementary material for: Zika Virus Infects Intermediate Progenitor Cells and Post-mitotic Committed Neurons in Human Fetal Brain Tissues
Source: Sci Rep. 2017 Nov 1;7:14883. doi: 10.1038/s41598-017-13980-2 (PMC5665882; doi:10.1038/s41598-017-13980-2)
Supplement: Supplementary file 1 — Supplementary Information [file 41598_2017_13980_MOESM1_ESM.pdf]

## SUPPLEMENTAL INFORMATION

# Zika Virus Infects Intermediate Progenitor Cells and Post-mitotic Committed Neurons in Human Fetal Brain Tissues

Ming-Yi Lin, Yi-Ling Wang, Wan-Lin Wu, Victoria Wolseley, Ming-Ting Tsai, Vladimir Radic,

Matthew E. Thornton, Brendan H. Grubbs, Robert H. Chow, I-Chueh Huang

| Donor | Age (GW) | Antibodies used in this work |
|---|---|---|
| 1 | 14.1 | Flavivirus E protein (millipore, MAB10216, mouse monoclonal, 1:500) |
| 2 | 14.4 | ZIKV E protein (GeneTex, GTX634155, mouse monoclonal IgG2b, 1:200) |
| 3 | 15.2 | Ki-67 (ThermoFisher, PA5-19462, rabbit polyclonal, 1:500) |
| 4 | 15.5 | SOX2 (Abcam, ab110145, goat polyclonal, 1:100) |
| 5 | 15.6 | NESTIN (millipore, ab5922, rabbit polyclonal, 1:200) |
| 6 | 16.2 | TBR2 (millipore, ab15894, chicken polyclonal, 1:200) |
| 7 | 16.6 | TBR2 (millipore, ab2283, rabbit polyclonal, 1:300) |
| 8 | 17.1 | TBR2 (millipore, MA5-24291, mouse monoclonal, 1:200) |
| 9 | 17.2 | SATB2 (Santa Cruz Biotechnology, sc-81376, mouse monoclonal IgG1, 1:250) |
| 10 | 17.3 | MAP2 (ThermoFisher, PA1-10005, chicken polyclonal, 1:1000) |
| 11 | 17.5 | GFAP (Abcam, ab7260, rabbit polyclonal, 1:400) |
| 12 | 17.6 | NG2 (millipore, ab5320, rabbit polyclonal, 1:25) |
| 13 | 17.6 | IBA1 (WAKO, 019-19741, rabbit polyclonal, 1:250) |
| 14 | 18.5 | CD11b (Abcam, ab8878, rat monoclonal, 1:200) |
| 15 | 18.5 | HLA-DR (Abcam, ab134038, rat monoclonal, 1:200) |
| 16 | 19 | Cleaved Caspase-3 (Cell Signaling, 9661, rabbit polyclonal, 1:100) |
| 17 | 19 | |
| 18 | 21 | |

**Extended Data Table 1. Human fetal brain tissues analyzed in our research.**
Gestational ages of donors and antibodies used for image studies were listed.

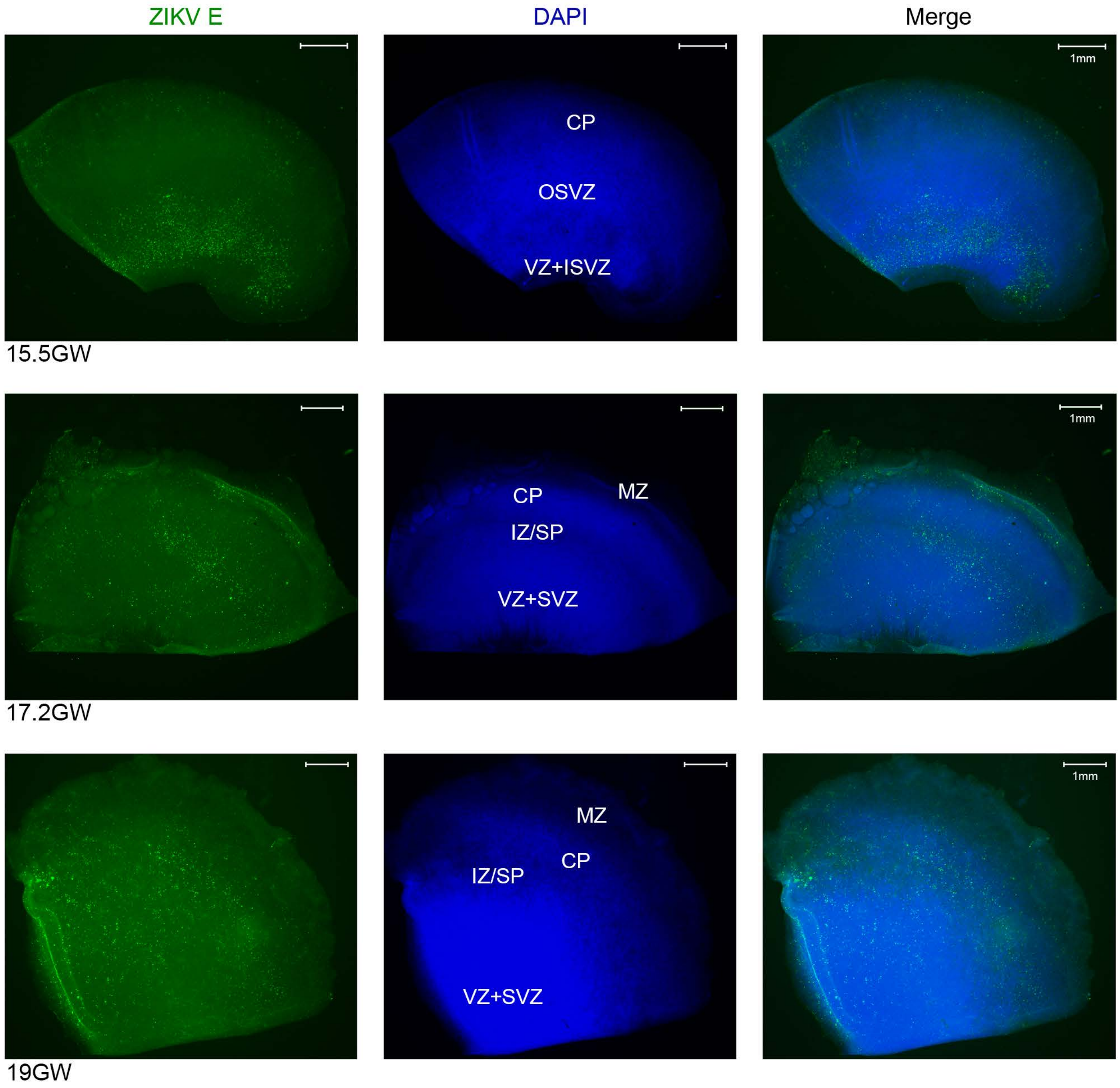


**Extended Data Figure 1 | ZIKV infection in human fetal brain tissue slices.** Experiments similar to those in Fig. 1a except that merged and single-color images of Fig. 1a are shown. (VZ: ventricular zone, ISVZ: inner subventricular zone, OSVZ: outer subventricular zone, IZ: intermediate zone, SP: subplate, CP: cortical plate, MZ: marginal zone.)

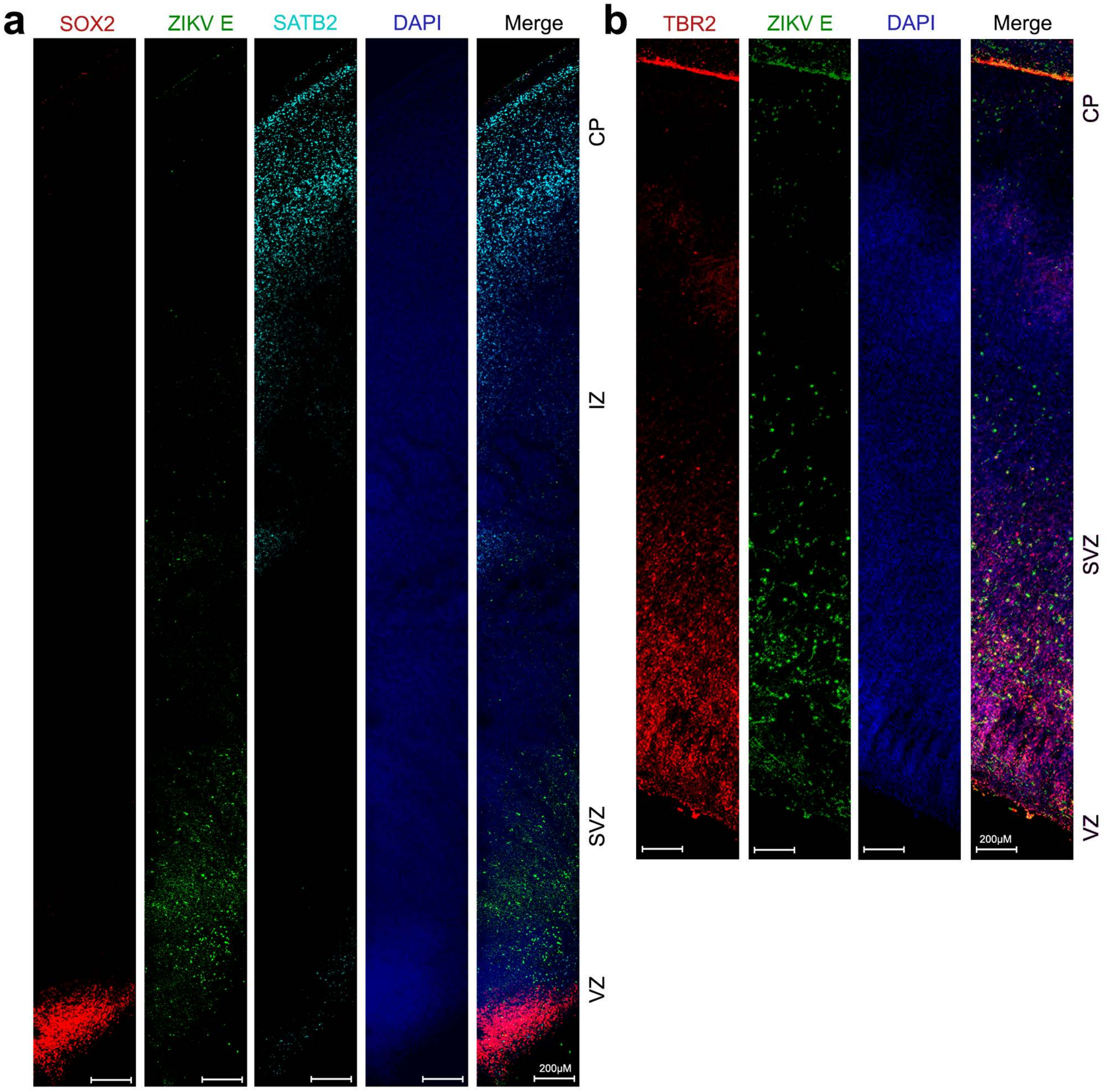


**Extended Data Figure 2 | ZIKV infection in human fetal brain tissue slices. a, b,** Human fetal brain tissues from donors at the indicated gestation ages were infected with 1x106 PFU of ZIKV strain MR766. Two days later, infected tissues were labeled with the indicated antibodies and imaged by confocal microscopy. (VZ: ventricular zone, SVZ: subventricular zone, IZ: intermediate zone, CP: cortical plate.)

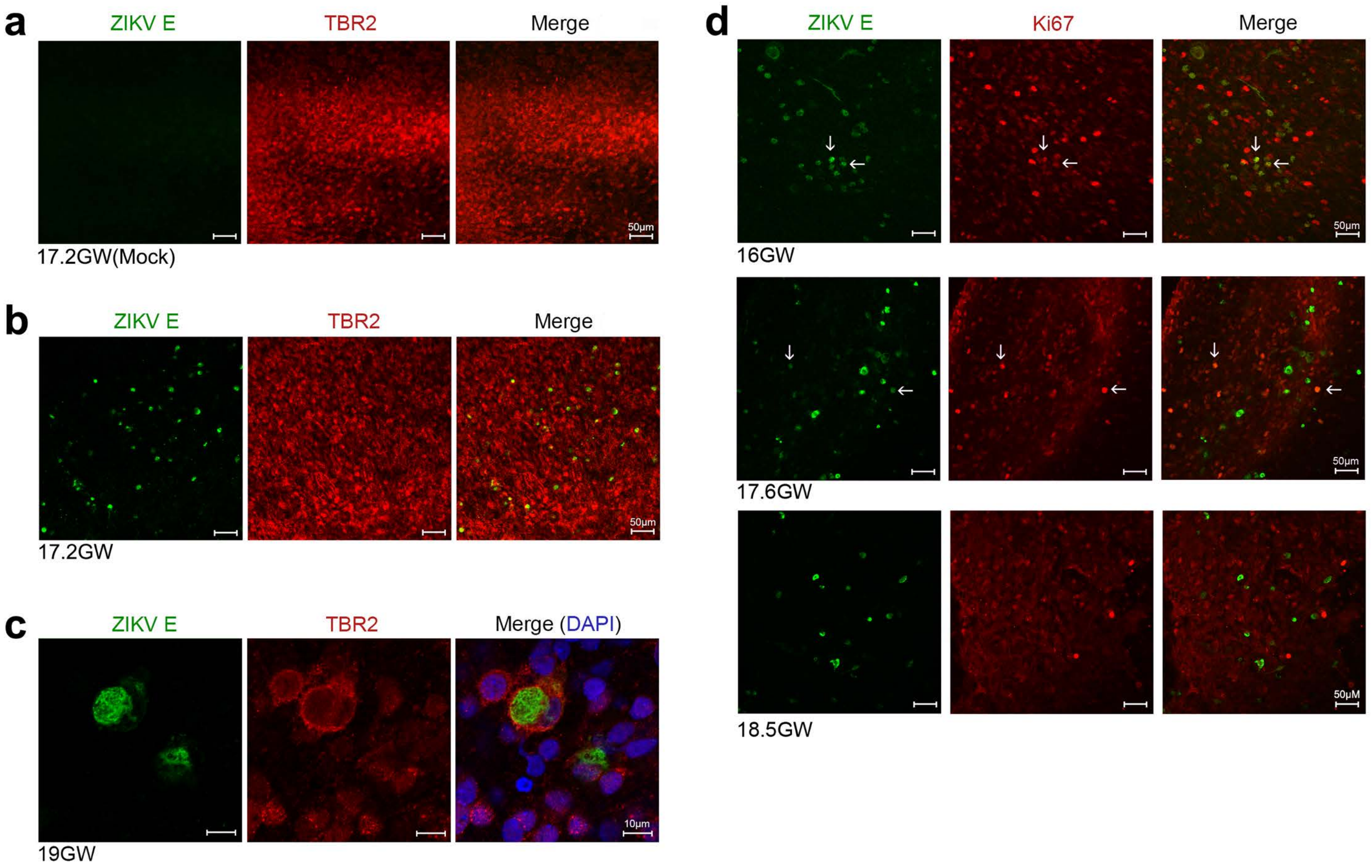


**Extended Data Figure 3 | ZIKV infects IPCs. a, b,** Experiments were similar to those in Fig. 1b except that images of uninfected controls (a) and representative images used for quantitative analysis in Fig. 1d (b) are shown. **c,** Single-color and merged x-y axis images of Fig. 1c are shown. **d,** Human fetal brain tissues from donors at the indicated gestation ages were infected with 1x106 PFU of ZIKV strain MR766. Two days later, infected tissues were labeled with the indicated antibodies. Labeled cells were imaged by confocal microscopy. Arrows indicate ZIKV-infected Ki67-positive cells.

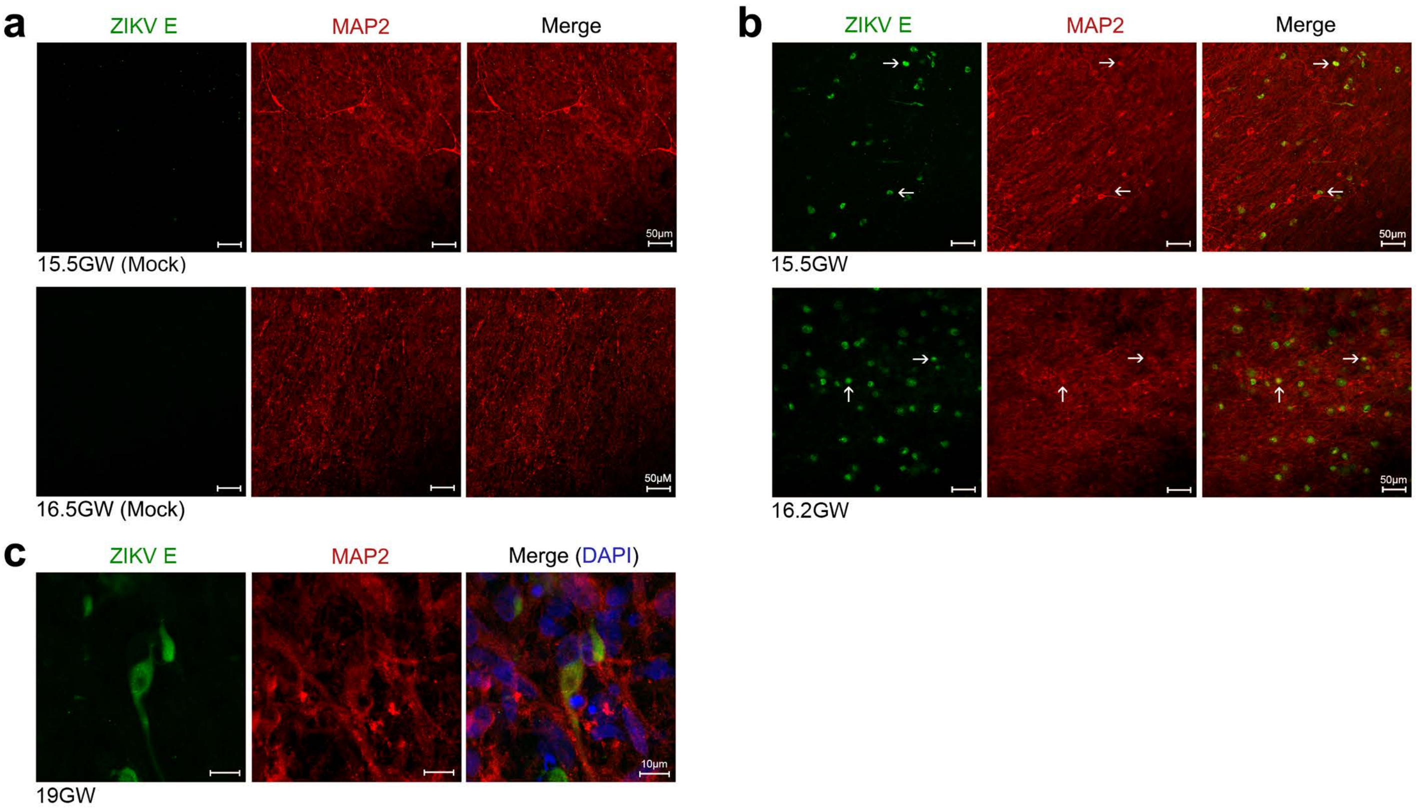


**Extended Data Figure 4 | ZIKV infects post-mitotic committed neurons. a, b,** Experiments were similar to those in Fig. 1e except that images of uninfected controls (a) and representative images used for quantitative analysis in Fig. 1g (b) are shown. Arrows indicate ZIKV-infected MAP2-positive cells. **c,** Single-color and merged x-y axis images of Fig. 1f are shown.

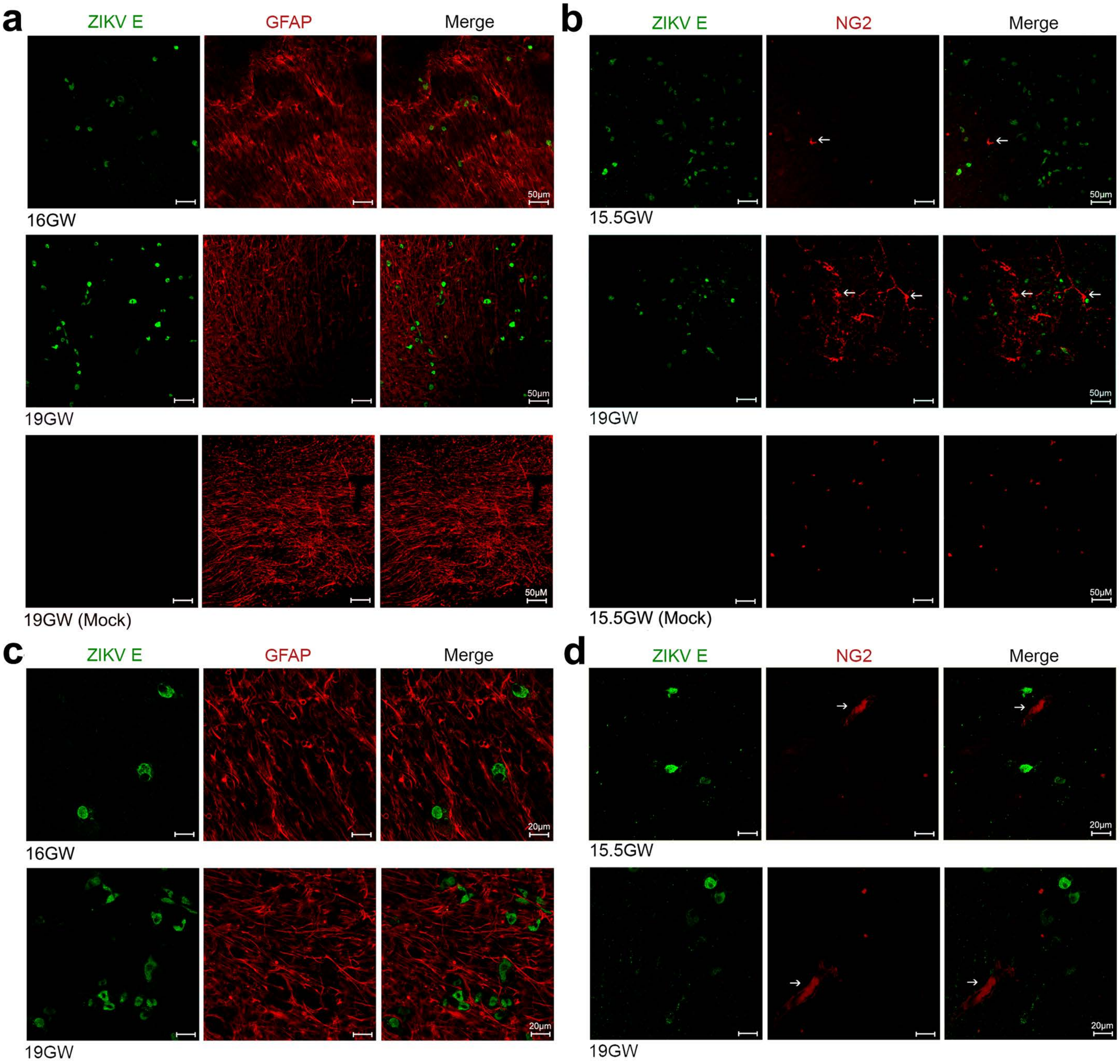


**Extended Data Figure 5 | GFAP- and NG2-positive cells are not major target cells for ZIKV. a, b,** Human fetal brain tissues from donors at the indicated gestation ages were mock infected or infected with 1x106 PFU of ZIKV strain MR766. Two days later, tissues were labeled with the indicated antibodies. Labeled cells were imaged by confocal microscopy. **c, d,** Experiments were similar to those in (a) and (b) except that high magnification images are shown. Arrows indicate oligodendroglial cells. 10-20 fields and more than 300 ZIKV-infected cells for each brain slice were inspected to draw our conclusions. Markers used included GFAP for neural stem cells or astroglial cells and NG2 for oligodendroglial cells.

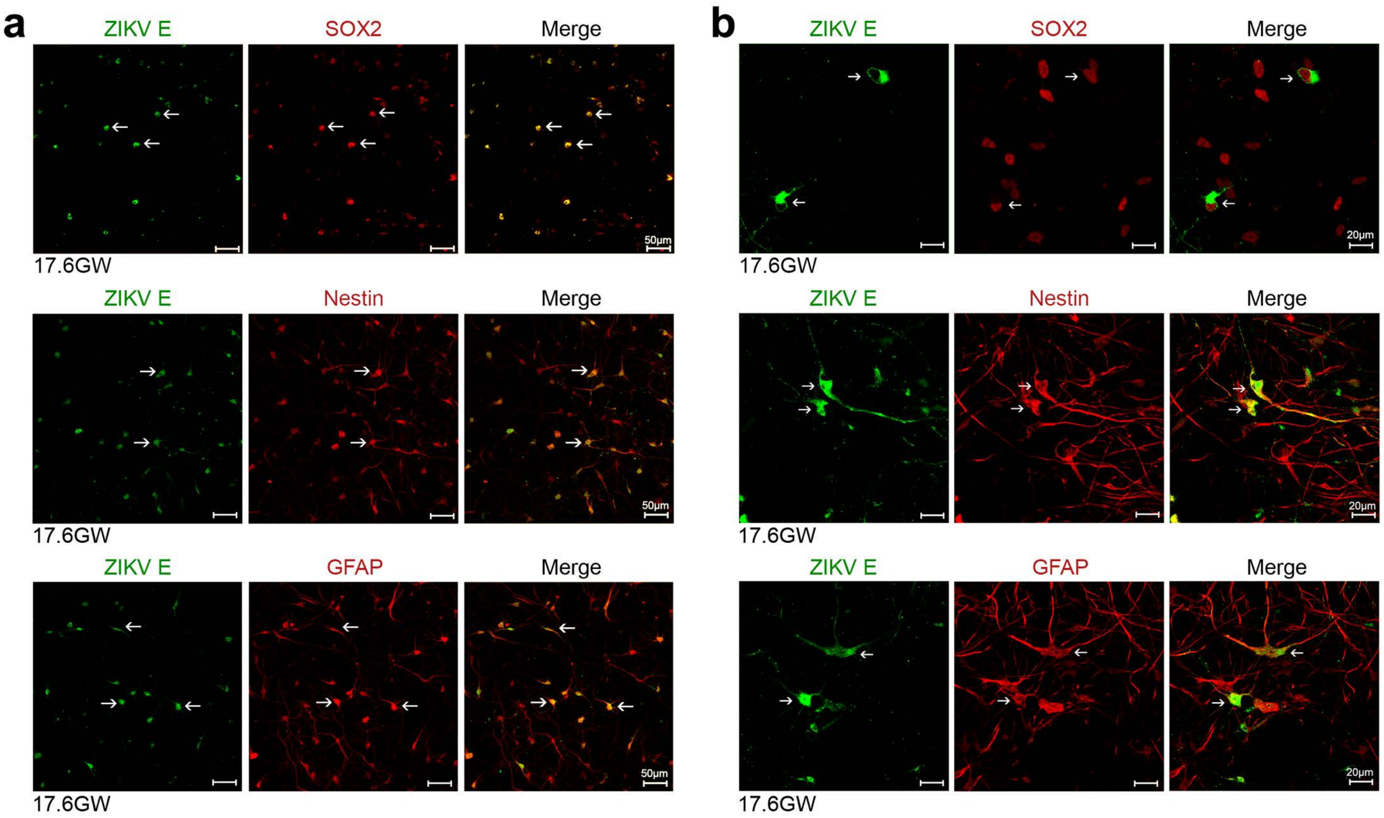


**Extended Data Figure 6 | ZIKV infects neural stem cells in monolayer cultures derived from human fetal brain tissues. a,** Brain monolayer derived from human fetal brain tissues from donors at the indicated gestation ages were infected with 1x106 PFU of ZIKV strain MR766. Two days later, infected cells were labeled with the indicated antibodies. Labeled cells were imaged by confocal microscopy. **b,** Experiments were similar to those in (a) except that high magnification images are shown. Arrows indicate ZIKV-infected cells. Markers used included SOX2 and nestin for neural stem cells, and GFAP for neural stem cells or astroglial cells.

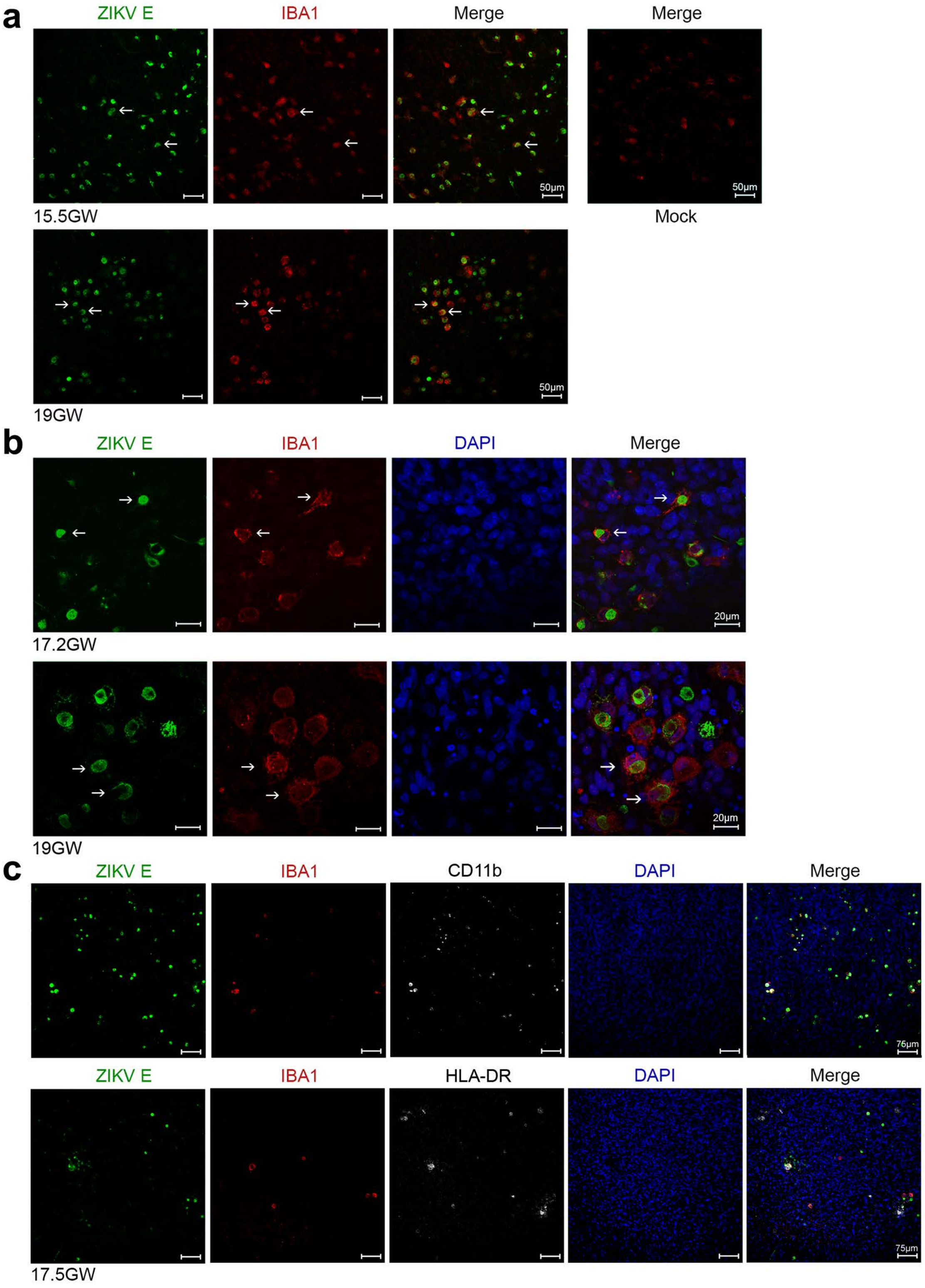


**Extended Data Figure 7 | ZIKV infection activates microglial cells. a-c,** Human fetal brain tissues from donors at the indicated gestation ages were infected with 1x106 PFU ZIKV strain MR766. Two days later, infected tissues were labeled with the indicated antibodies. Labeled cells were imaged by confocal microscopy.

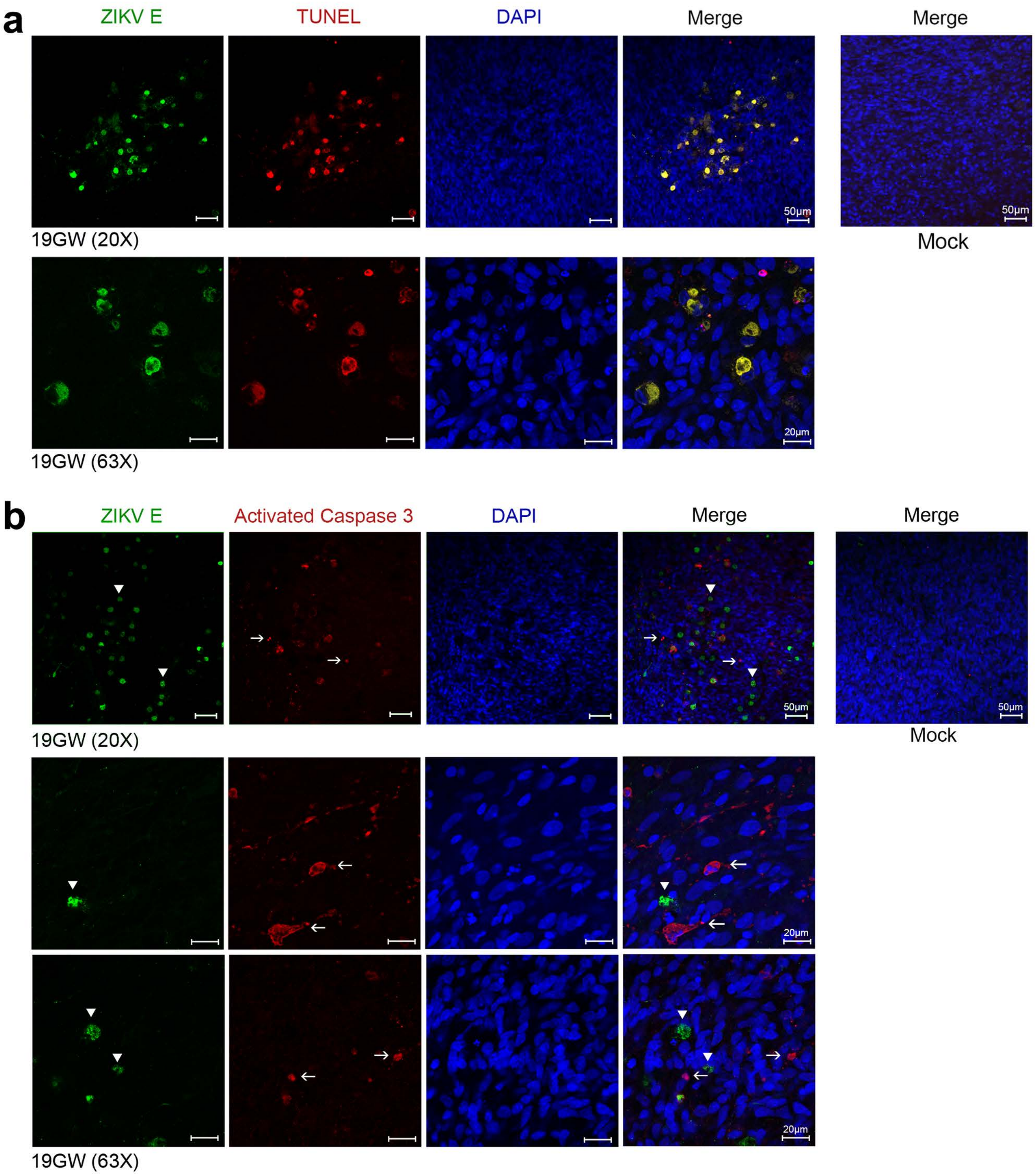


**Extended Data Figure 8 | ZIKV infection induces DNA fragmentation and apoptosis in cells. a,** Experiments were similar to those in Fig. 3c except additional lower magnification images and single-color images of Fig. 3c are shown. **b,** Experiments were similar to those in Fig. 3d except additional lower and higher magnification images and single-color images of Fig. 3d are shown. Arrowheads indicate ZIKV-infected cells and arrows indicate uninfected cells.

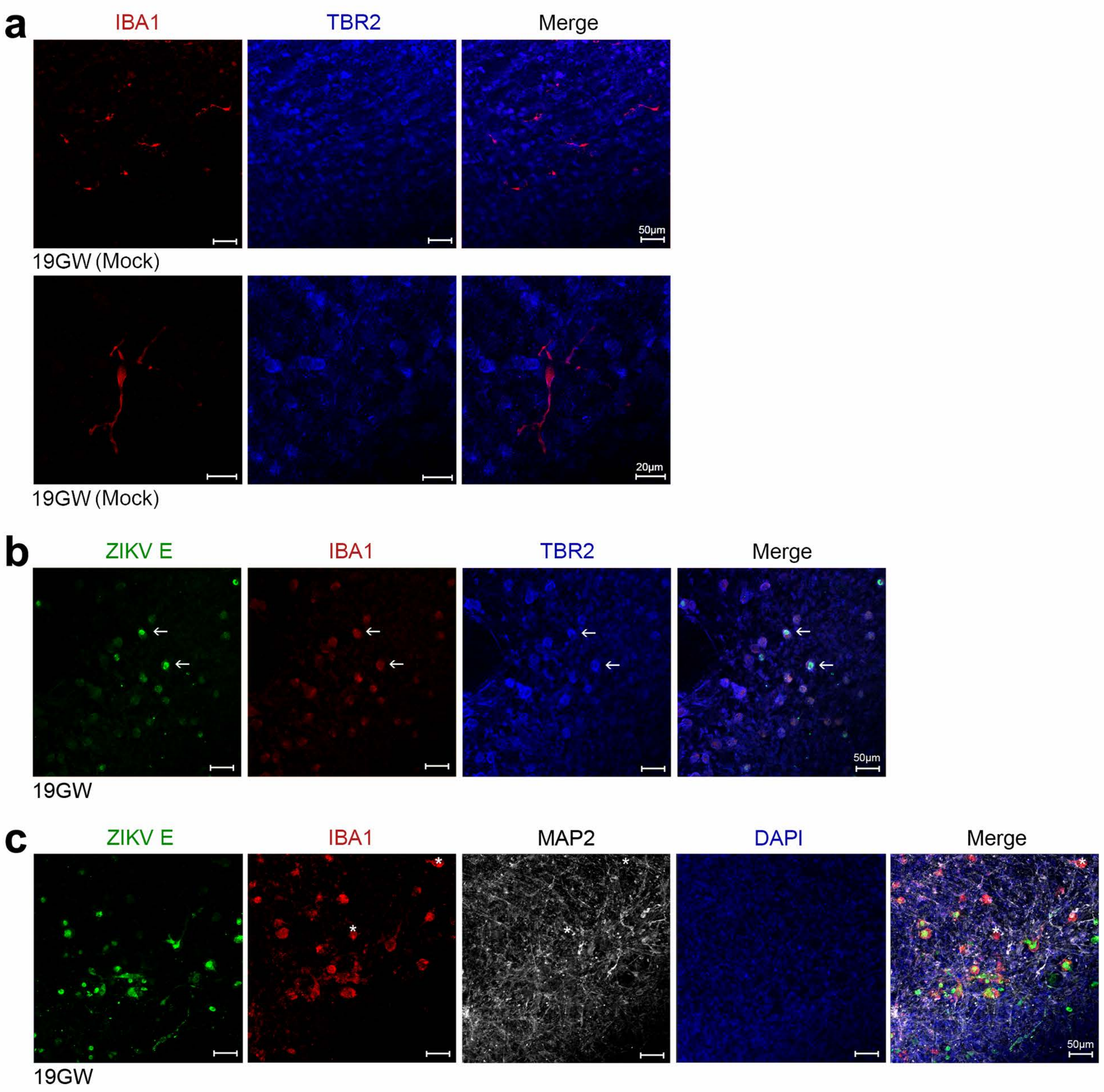


**Extended Data Figure 9 | ZIKV infection activates microglial cells. a,** Human fetal brain tissues from donors at the indicated gestation ages were labeled with the indicated antibodies. There was no cross-reaction between Tbr2 and Iba1 antibodies used in our studies. **b,** Experiments were similar to those in Fig. 3e except that lower magnification images are shown. Arrows indicate colocalization of Tbr2 and Iba1 in ZIKV-infected cells. **c,** Experiments were similar to those in Fig. 3f except that lower magnification images are shown. * indicates Iba1-positive but MAP2-negative cells.
